# RAPID IMPLEMENTATION OF REAL-TIME REVERSE-TRANSCRIPTION POLYMERASE CHAIN REACTION (REAL-TIME RT-PCR) ASSAY FOR THE DETECTION OF SARS-COV-2 IN A MOROCCAN HOSPITAL

**DOI:** 10.21010/Ajid.v16i2S.8

**Published:** 2022-08-17

**Authors:** Belefquih Bouchra, Maher Wissal, Cheikh Amine, Hamdani Wail, Zaid Younes, Kabbaj Hakima, Seffar Meryem, Dakka Hanae, Hadami Khaoula, Allaoui Afaf, Benouda Amina

**Affiliations:** 1 Biolife Medical Analysis Laboratory, Harhoura, Temara, Morocco; 2 Microbiology Laboratory, Cheikh-Zaid University Hospital, Abulcasis University of Health Sciences, Rabat, Morocco; 3 Central Virology Laboratory, Ibn Sina University Hospital Center, Rabat Morocco; 4 Laboratory of Biology of Human Pathologies, Faculty of Sciences, Mohammed V University, Rabat, Morocco

**Keywords:** Covid-19, SARS-Cov-2, molecular diagnostic, real-time RT-PCR, Morocco

## Abstract

**Background:**

The main challenge faced in the African countries was to implement efficient molecular diagnostic facilities and start Covid-19 diagnosis as fast as possible to handle the rapid and unpredictable rise of cases.

**Materials, Methods and Results:**

We describe our experience in implementing a molecular biology unit at Sheikh Zaïd International University Hospital in Rabat, with a delay as short as one week, and starting real-time RT-PCR assay for the detection of SARS-Cov-2 infection, since the outbreak widened in Morocco in mid-March, 2020.

**Conclusion:**

The challenges encountered in the first period of Covid-19 pandemic are still present. This work aims to give an example of a rapid and adaptive response in order to maintain our diagnosis ability for Covid-19 and for other pathogens.

## Introduction

By early December 2019, the first cases of severe acute respiratory failure, due to an unknown virus, appeared in Wuhan, China (Wang, 2020; Zhu, 2020). In January 2020, a novel coronavirus-named “severe acute respiratory syndrome coronavirus-2” (SARS-CoV-2) - was identified as the causative agent of suspicious pneumonia cases (Gorbalenya, 2020). By the end of January 2020, the World Health Organization (WHO) declared coronavirus disease 2019 (Covid-19) a public health emergency of international concern (WHO, 2020). In March 2020, Covid-19 outbreak was declared a global pandemic (Cucinotta, 2020). On the 21^st^ April 2020, 2,356,414 confirmed cases and 160,120 deaths in more than 200 contries, were linked to the virus (The Global Fund, COVID-19 Situation Report, 2020).

In Morocco, the first Covid-19 case was confirmed on 2^nd^ March 2020. Since then, the disease has spread throughout the national territory; as of April 2020, the number of cases had reached the threshold of 1000 positive cases (Ministry of Health, The Official Portal of Coronavirus in Morocco, 2020).

In such global pandemic scenarios, confirmation or elimination of suspected cases is critical, notably when clinical symptoms are difficult to distinguish from similar respiratory infections caused by other viruses or pathogens. Hence, molecular diagnosis is required for the detection of the virus.

During the early international spread of Covid-19 outbreak, researchers from all over the world tried to respond to the urgent need for such tests, and many molecular diagnostic assays have been made available, especially those based on SARS-CoV-2 RNA detection by real-time reverse-transcription polymerase chain reaction [real-time RT-PCR] (Centers for Disease Control and Prevention (CDC) 2020; Corman, 2020; Pfefferle, 2020).

The implementation, configuration, execution, and interpretation of this kind of molecular test in diagnostic laboratories, as well as the processing of a large numbers of samples, require both molecular biology infrastructures and a high degree of human interaction. However, in low-income countries, diagnostic laboratories face many challenges, including shortcoming of adapted settlement to molecular biology, pressure over supply chains of disposables, reagents and personal protection equipment (PPE), and lack of molecular biology trained staff.

In this study, we report our experience about the introduction and validation of SARS-CoV-2 real-time RT-PCR protocol at the molecular biology unit of the Sheikh Zaïd International University Hospital in Rabat. Thereby, we aimed at optimizing SARS-CoV-2 screening workflow according to WHO recommendations (WHO, Regional Office for South-East Asia 2020; WHO, 2020).

## Materials and Methods

### Assay design and recruitment of samples and controls

We could not afford waiting to bring SARS-Cov-2 cell culture supernatants from foreign suppliers to validate SARS-Cov-2 viral RNA detection procedure. Therefore, we chose an inter-laboratory comparison and validation approach that we carried out in two stages:

At the first stage of validation, we collected five positive RNA eluates of nasopharyngeal swabs of Covid-19 patients from national reference virology laboratories: the Laboratory of Virology of Mohammed V Military Teaching Hospital of Rabat and the Central Laboratory of Virology of In Sina University Hospital Center of Rabat. We also tested three stored (at -70°C) SARS-Cov-2 negative nasopharyngeal swab samples from patients tested before December 2019 at Sheikh Zaïd International University Hospital, for respiratory viruses; one of them had Influenza virus A (H1N1) RNA detected and two of them were negative.

We selected the envelope (E) and RNA-dependent RNA polymerase (RdRp) genes as PCR targets based on the protocol developed by the German Consultant Laboratory for Coronaviruses (Charité, Berlin) [Corman 2020].

Positive and negative controls were included for viral RNA detection. Positive control material consisted of synthetic Wuhan coronavirus 2019 E gene control (ref. 026N-03866) for E gene and SARS-CoV Frankfurt 1 ARN (ref. 004N-02005) for RdRp gene, which we ordered from the European Virus Archive (EVAg). Negative control was nuclease free distilled water (NTC). PCR efficiency was evaluated by making a ten-fold serial dilution for the positive controls (1:10, 1:100).

The second stage of our validation procedure is three *weeks* of double testing of 42 patient samples simultaneously in our laboratory and in the National Institute of Hygiene Virology laboratory, which was one of the three first authorized public laboratories to perform SARS-Cov-2 RT-PCR diagnosis. The samples were nasopharyngeal swabs in viral transport media (VTM) or bronchoalveolar liquid for intensive care patients, hospitalized at the Sheikh Zaïd International University Hospital.

The use of samples for diagnostic workflow optimization was approved by the local institutional review board represented by “Cheikh Zaid Foundation Ethics Committee”, under the document reference number “CEFCZ/PR/2020 (PR04)”.

### SARS-CoV-2 RNA isolation procedure

Sample preparation was carried out under a safety class 2 cabinet, wearing FFP2 filter masks while following the WHO recommendations (WHO, 2020).

We worked with several commercial extraction kits according to the market availability. So, viral RNA extraction was performed based on magnetic bead separation technology, using MagPurix® Viral Nucleic Acid Extraction Kit ZP02003 - Zinexts and MagMAX™ Viral/Pathogen Nucleic Acid isolation kit A42352 – Applied Biosystems, or based on silica membrane technology, by using NucleoSpin™ Mini Kit - Macherey Nagel and QIAamp® viral RNA Mini kit - Qiagen. All extractions were carried out according to the manufacturer’s instructions.

### SARS-CoV-2 RNA detection

Choice criteria for SARS-CoV-2 real-time RT-PCR protocol were mainly WHO recommendations (WHO, 2020) with reliable published validation protocols (Corman, 2020; Konrad, 2020) and rapid availability of reagents.

The targeted viral genes were E gene for Sarbecovirus assay as first-line screening and RdRp gene for SARS-CoV-2 specific assay as SARS-CoV-2 confirmatory screening. Primer-probe sets were chosen and ordered from Tib-MolBiol as described by Corman et al. (2020), with the use of SARS-CoV-2 specific probe RdRP-SARSr-P2 - Table 1.

**Table 1:** SARS CoV-2 real-time RT-PCR primers and probe sequences used in this study accordingly to Corman et al.(2020).

Target Gene	Primers and probes Sequence (5’-3’)
E gene (Screening)	E-Sarbeco-F : ACAGGTACGTTAATAGTTAATAGCGT
	E-Sarbeco-R : ATATTGCAGCAGTACGCACACA
	E-Sarbeco-P : FAM-ACACTAGCCATCCTTACTGCGCTTCG-BBQ
RdRpgene (Confirmatory)	RdRp-SARSr-F : GTGARATGGTCATGTGTGGCGG
	RdRp-SARSr-R : CARATGTTAAASACACTATTAGCATA
	RdRp-SARSr-P : FAM-CAGGTGGAACCTCATCAGGAGATGC-BBQ

FAM: 6-carboxyfluorescein; BBQ: blackberry quencher.

Real time RT-PCR was initially performed with the QuantiTect Virus + ROX Vial kit (QIAGEN) on the Rotor-Gene Q Real-Time PCR Detection System (QIAGEN), using Green channel for reading. Since we had a supply problem for RT-PCR Master Mixes, we had to adapt PCR protocol for four different reagents *during the first month of establishment* (Takyon™ two-step RT-PCR mix (Eurogentec), Takyon™ one-step RT-PCR mix (Eurogentec), LightCycler Multiplex RNA Virus Master (Roche) and SuperScript™ III Platinum™ RT-PCR mix (Invitrogen)); cycling conditions are presented in Table 2. Moreover, each time we had to pass the standards dilution ranges, two negative eluates and up to three SARS-CoV-2 positive eluates. Primers and probes were however the same for all RT-PCR mixes.

**Table 2:** RT-PCR kits used in this study with their corresponding cycling protocols

RT-PCR kits	Cycling conditions	
QuantiTect Virus +ROX Vial kit (QIAGEN)	RT step, 50°C for 20 min	
	Activation, 95°C for 5 min	
	Denaturation, 95°C for 15 sec	} X 45 cycles
	Annealing/Extension, 60°C for 30sec	
Takyon RT-PCR mix (Eurogentec)	RT step, 48°C for 10 min	
	Activation, 95°C for 3 min	
	Denaturation, 95°C for 15sec	} X 45 cycles
	Annealing/Extension, 60°C for 30sec	
LightCycler Multiplex RNA Virus Master (Roche)	RT step, 48°C for 10 min	
	Activation, 95°C for 30sec	
	Denaturation, 95°C for 5sec	} X 45 cycles
	Annealing/Extension, 60°C for 30sec	
SuperScriptIII Platinum RT-PCR mix (Invitrogen)	RT step, 55°C for 10 min	
	Activation, 95°C for 3 min	
	Denaturation, 95°C for 15sec	} X 45 cycles
	Annealing/Extension, 60°C for 30sec	

## Results

### First part of validation

#### PCR efficiency evaluation

**Figure 1:**
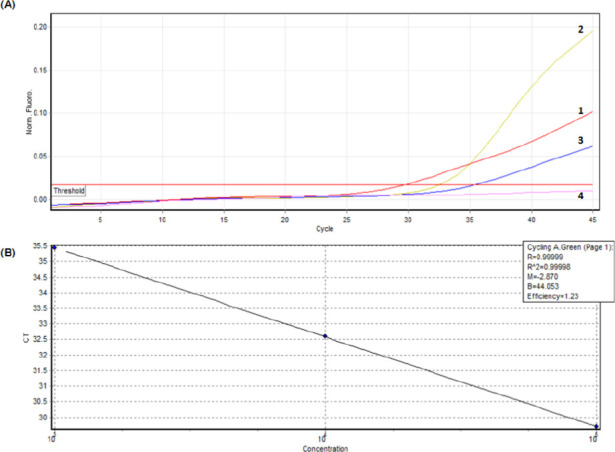
PCR results of E gene PC dilution series. (A) Amplification plots of (1) pure PC at 100,000 copies/ml, (2) 1:10 PC dilution at 10,000 copies/ml, (3) 1:100 PC dilution at 1000 copies/ml and (4) no amplification for NTC. (B) Standard curve made by PC dilution.

All standard serial dilutions made for efficiency evaluation of PCRs performed with the different Master Mixes showed satisfactory analysis for both E and RdRp genes. Figure 1 represents the real-time RT-PCR results of the serial dilution made for E gene positive control (PC) performed with the QuantiTect Virus +ROX Vial kit on the Rotor-Gene Q, in which a NTC was included.

The limit of detection for E gene was fixed at 36 amplification cycles (CT, cycle treshold), corresponding to about 500 copies/ml. Thus, a CT of 36 was set to discriminate between positive and negative samples for E gene.

### Positive and negative eluates from reference virology laboratories

Clinical samples were declared positive or negative following the interpretation presented in Table III.

**Table 3:** SARS-CoV-2 real-time RT-PCR results interpretation based on E and RdRp gene detection

Target gene	Sample	Positive control	Negative control	Interpretation
E	- (CT>36)	+ CT [28-36]	-	Negative
RdRp	-	+ CT [28-38]	-	
E	- (CT>36)	-	-	Invalid ; Repeat
RdRp	-	-	-	
E	+	+ CT [28-36]	-	SARS-CoV-2
RdRp	+	+ CT [28-38]	-	
E	+	+ or- CT [28-36]	+	Contamination ; Repeat
RdRp	+	+ or -CT [28-38]	+	
E	+ (CT<36)	+ or - CT [28-36]	-	Betacoronavirus
RdRp	-	+ CT [28-38]	-	
E	-	+ or - CT [28-36]	-	Not reliable ; Repeat
RdRp	+	+ CT [28-38]	-	

Five SARS-Cov-2 positive eluates were found positive with detection of both E gene and RdRp gene in our laboratory and three negative samples were found negative for SARS-Cov-2 RNA.

### Interlaboratory comparison

Five out of the 42 samples (11.9%) had discordant results: one was positive in our laboratory but negative in the reference laboratory (RL), two were doubtful in our laboratory with negative results in RL, and two were negative in our laboratory but positive or doubtful in RL. PCR results concordance between the two laboratories was assessed by calculating Cohen’s kappa coefficient (K), see Table IV.

**Table 4:** Interlaboratory comparison of SARS-CoV-2 real-time RT-PCR results

	Our lab positive or doubtful	Our lab negative	Kappa
Reference lab positive or doubtful	18	2	0.81
Reference lab negative	3	20	

## Discussion

Covid-19 pandemic have challenged the ability of rapid preparedness and adaption at all scales, including diagnosis laboratories within the context of supply chain pressure and fast changing management procedures.

In the reported experience, we demonstrate the need for timely adaptation to available reagents and machines. The lack of reference reagents and consumables was a major challenge facing our laboratory during this crisis; we had to use five different master mix brands for the in-house Covid-19 RT-PCR and lately in-vitro diagnostic (IVD) RT-PCR kits, when they were available. Besides, we changed RNA extraction procedure more than four times, going from manual procedure to automatic procedure and vice versa.

Fortunately, the pressure on the supply chain slowed when we had to face the mass screening demand. Other challenges came with large demand on RT-PCR testing. Workflow organization, information system issues, and reliable pooling procedures were big concerns.

We also share a proposal of a rapid and continuous validation method for the implemented techniques. Waiting for the culture supernatant of SARS-CoV2 wasn’t possible regarding lockdown conditions and complicated procedures. We chose to use the positive control supplied by the same providers of primers and probes to perform the first step of validation, we then performed double testing to be more confident with our laboratory results. The five discordant results have been controlled at least three times each, they appear to be positive patients in 3 cases and negative in two. The three positive patients had a clinical presentation and chest tomography data in favor of Covid-19.

We decided to repeat PCR testing in patients with Covid-19 symptoms. Currently, we are still facing the same challenges as early pandemic phases, supply chain are under pressure, we are still obliged to adapt and change the used reagent and protocols according to their availability. The rise of war in Europe is not helping our issues. This study hopes to give an example of adaptation procedure in order to ensure continuity and efficiency of our molecular diagnosis facilities.

### Conflict of interest:

The authors declare that there is no conflict of interest associated with this study.

**List of Abbreviations:**
Covid-19: coronavirus disease 2019,
CT: cycle threshold,
E: envelope,
EVAg: European Virus Archive,
IVD: in-vitro diagnostic,
K: Cohen’s kappa coefficient,
NTC: nuclease free distilled water,
PC: positive control,
PPE: personal protection equipment,
RdRp: RNA-dependent RNA polymerase,
RL: reference laboratory,
RT-PCR: reverse-transcription polymerase chain reaction,
SARS-CoV-2: severe acute respiratory syndrome coronavirus 2,
VTM: viral transport media,
WHO: World Health Organization
